# Effects of a 10-week musical instrument training on cognitive function in healthy older adults: implications for desirable tests and period of training

**DOI:** 10.3389/fnagi.2023.1180259

**Published:** 2023-08-15

**Authors:** Xueyan Wang, Takahiro Soshi, Masatoshi Yamashita, Marcelo Kakihara, Takanobu Tsutsumi, Shoko Iwasaki, Kaoru Sekiyama

**Affiliations:** ^1^Graduate School of Advanced Integrated Studies in Human Survivability, Kyoto University, Kyoto, Japan; ^2^Center for Genomic Medicine, Graduate School of Medicine, Kyoto University, Kyoto, Japan

**Keywords:** healthy aging, musical instrument training, intervention study, verbal working memory, randomized controlled trial, test-retest practice effect

## Abstract

**Introduction:**

Previous studies have shown that musical instrument training programs of 16 or more weeks improve verbal memory (Logical Memory Test delayed recall), processing speed (Digit Symbol Coding Test), and executive function (Trail Making Test Part B) of musically untrained healthy older adults. However, it is unclear whether shorter-period instrument training can yield similar effects. We sought to (1) verify those results and (2) clarify if intervention effects could be detected using other measures such as reaction time.

**Methods:**

Healthy older adults (mean age = 73.28 years) were pseudo-randomly assigned to an untrained control group (*n* = 30) or an intervention group (*n* = 30) that received a weekly 10-session musical instrument training program (using melodica). We conducted neuropsychological tests on which intervention effects or association with musical training were reported in previous studies. We newly included two reaction time tasks to assess verbal working memory (Sternberg task) and rhythm entrainment (timing task). Intervention effects were determined using a “group × time” analysis of variance (ANOVA).

**Results:**

The intervention effects were detected on the reaction time in Sternberg task and phonological verbal fluency. Although intervention effects had been reported on Logical Memory test, Digit Symbol Coding Test and Trail Making Test in previous studies with longer training periods, the present study did not show such effects. Instead, the test-retest practice effect, indicated by significant improvement in the control group, was significant on these tests.

**Discussion:**

The present results indicated the usefulness of working memory assessments (Verbal Fluency Test and Sternberg task) in detecting the effects of short-term melodica training in healthy older adults. The practice effect detected on those three tasks may be due to the shorter interval between pre- and post-intervention assessments and may have obscured intervention effects. Additionally, the findings suggested the requirement for an extended interval between pre- and post-tests to capture rigorous intervention effects, although this should be justified by a manipulation of training period.

## 1. Introduction

Active lifestyle, characterized by engagement in leisure activities, has been associated with slower cognitive decline in healthy older adults (Scarmeas and Stern, 2003). Longitudinally, healthy older adults are reported to improve their cognition after participating in short-term interventions; for example, physical activity training such as dancing (Kosmat and Vranic, 2017; Esmail et al., 2020) and computerized cognitive training (Simon et al., 2018; ten Brinke et al., 2018, 2020). Playing musical instrument, having both cognitive and motor training aspects, is also reported as one of the leisure activities which reduces the risk of dementia in an epidemiological study (Verghese et al., 2003). There is increasing attention to the impact of music on brain and cognitive function in older adults in recent years (Cuddy et al., 2020). In cross-sectional studies, compared with age-matched non-musicians, older musicians have been found to have not only superior music performance skills but also reserve related cognitive functions. Their life-long musicianship has been associated with better reserved auditory processing (Parbery-Clark et al., 2011; Zendel and Alain, 2012; Amer et al., 2013), executive function (Hanna-Pladdy and MacKay, 2011; Moussard et al., 2016; Yamashita et al., 2022), and verbal memory (Hanna-Pladdy and Gajewski, 2012; Fauvel et al., 2014). Although cross-sectional studies have associated music playing with positive outcomes in cognitive ability (Schneider et al., 2019), the advantages in older musicians are not always found over non-musicians (Dittinger et al., 2019). Moreover, the causal relationship remains unclear; thus, intervention studies are needed.

The effects of music training interventions on cognitive function have been studied primarily in children. These studies have shown that music training interventions can promote the development of auditory processing ability (Habibi et al., 2016; Barbaroux et al., 2019), language ability (Ho et al., 2003; Linnavalli et al., 2018; Nan et al., 2018; Barbaroux et al., 2019), executive function (Roden et al., 2014a,b; Habibi et al., 2018; Hennessy et al., 2019; Bugos et al., 2022), and verbal working memory (Rickard et al., 2010; Guo et al., 2018). Contrarily, studies on music training interventions in healthy older adults are still limited, although several studies have examined music therapy for pathological aging (Clair and Memmott, 2008; Ridder et al., 2013; Chu et al., 2014) and rehabilitation after brain injury (Sihvonen et al., 2017; Vik et al., 2018, 2019).

One pioneering intervention study investigated the effects of musical instrument training program in healthy older adults with no formal music training (Bugos et al., 2007). In that randomized controlled trial (RCT) study, participants in the intervention group (IG) received individualized piano instructions for six months and were compared with participants in the control group (CG), who had received no training. The IG significantly improved their performance on Trail Making Test (TMT) Part B and Digit Symbol Coding Test (DSCT) compared with the CG, which suggests an effect of participation in musical instrument training on executive function and processing speed. In a non-RCT study, participants in a four-month group piano training significantly improved their performance on the Stroop task (Stroop Color subtest and Stroop Color Word subtest) compared with those engaged in other leisure activities, reflecting enhanced selective processing and inhibitory control following the training program (Seinfeld et al., 2013).

Our research team also tested healthy older adults in an RCT study to investigate the effects of instrument (keyboard harmonica: melodica) training on cognitive function. The IG received a 16-week grouped training program and significantly improved their delayed story recall (in Logical Memory Test of Wechsler Memory Scale: LMT) compared with the CG participants who were instructed to maintain their usual leisure activities (Guo et al., 2021). However, unlike training by playing the piano (Bugos et al., 2007; Seinfeld et al., 2013), our melodica program did not detect improvement in executive function or processing speed, but only in delayed story recall.

In sum, RCT studies of musical instrument training programs of 16 weeks or more (1 session per week) have shown improvement on DSCT, TMT Part B, and LMT delayed recall in healthy older adults. This study sought to determine whether a shorter period of instrument intervention could also improve cognitive function in healthy older adults. Such information is useful for planning intervention programs in local governments where the whole program should be completed in a fiscal year. Additionally, prior studies have shown that other types of general mental stimulation interventions improve healthy older adults’ cognitive functions after a shorter period (Kelly et al., 2014). Theater art interventions improved the cognition of healthy older adults within 8 sessions in 4 weeks (Noice et al., 2004; Noice and Noice, 2008). Cognitively stimulating leisure activities showed effects after 10 training sessions in 4 months (Tesky et al., 2011). Healthy older adults also improved their reasoning ability after 10 sessions of intervention (Margrett and Willis, 2006). Taken together, healthy older adults showed their potential to improve the cognition through general mental stimulation intervention within 10 sessions. Thus, this study investigated whether a 10-week melodica training intervention could improve healthy older adults’ cognitive function similarly to longer-term programs.

We aimed to conduct a randomized controlled study with pre-test/post-test measurements to (1) determine if a 10-week intervention can replicate the improvements in cognitive functions reported in previous longer-term instrument training programs in healthy older adults and (2) determine if the effect of the training can be detected using other assessments, such as reaction time (RT) measurements which may be more sensitive due to wider variation among different participants, when the effect is subtle.

## 2. Materials and methods

### 2.1. Participants and setting

Participants were recruited in Yao, Osaka, Japan using the following criteria: aged 60 years and older, community-dwelling, without previous formal musical training, willing to participate in group melodica training for 10 weeks, and two cognitive test sessions pre- and post-intervention. The recruitment activities, which took place between June and September of 2019, included distributing a printed brochure to every household in the community, holding briefings for the project, and passing on information via word of mouth. Eighty Japanese older adults (67 females) between 61 and 87 years of age participated in this study.

To investigate only healthy older adult novices, we used a self-report daily condition questionnaire and neuropsychological assessments to exclude individuals who met the exclusion criteria of having any of the following: a history of head injury (*n* = 3); use of psychotropic medication (*n* = 3); Mini-Mental State Examination (MMSE) of less than 26 points (*n* = 0); LMT delayed recall of more than 2 SD less than the age-appropriate mean (Kawano, 2012) (*n* = 0); experience of musical instrument training for at least 5 years (not including musical training as part of elementary and secondary education) (*n* = 9) (Bugos et al., 2007; Sadakata and Sekiyama, 2011; Zhang et al., 2020). Additionally, five participants quit the study after the baseline assessment. As a result, 60 participants were included in data analysis.

### 2.2. Procedures

A self-report questionnaire and the baseline cognitive function pre-tests were administered over 2 weeks in September 2019. Cognitive function was measured by using software that we developed in-house, the presentation of pre-recorded instructions and time measurements were computed for the test procedure. All the instructions, read by a female native Japanese speaker, were recorded in advance using a linear pulse code modulation recorder. During the test, the examiner played each instruction through a loudspeaker connected to a personal computer. The entire test process was recorded using a digital voice recorder (IC recorder) for scoring and future confirmation with the permission of each participant. Each participant took the tests individually in a quiet room for approximately 70 min after answering the questionnaires. Participants were permitted to take a brief midway rest, if necessary. The test battery to measure cognitive function is described in section “2.4. Cognitive function tests and questionnaires.”

**FIGURE 1:**
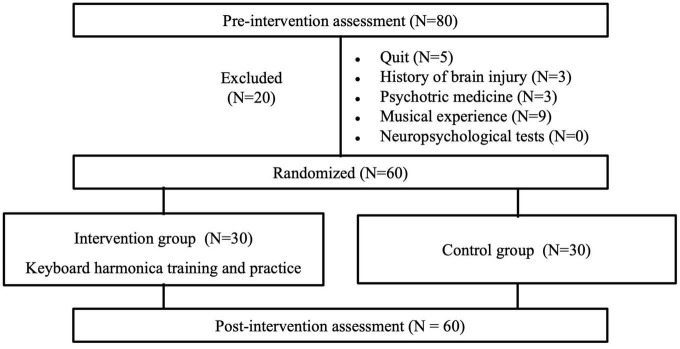
Flow chart of distribution of participants throughout the study.

After excluding those deemed ineligible, the IG and CG were pseudo-randomly assigned by checking that the two groups were not significantly different in each cognitive test at baseline (see section “3. Results”). As Figure 1 shows, 60 participants met the inclusion criteria and were assigned to either the IG (*n* = 30) or CG (*n* = 30). We informed the participants of the assignment results through telephoning. The IG received the 10 weekly sessions of melodica group training intervention, while the CG maintained their usual life habits and practices. The post-tests were administered following the last intervention session in December. The study design was approved by the ethics committee of Kyoto University (29-P-7). All participants provided written informed consent.

### 2.3. Intervention program

**FIGURE 2:**
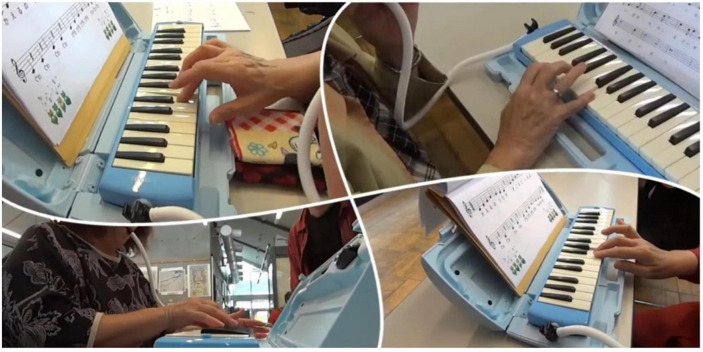
Musical instrument training session. The melodica (keyboard harmonica) was used for the training session.

The intervention consisted of 10 weekly sessions of 70-min melodica group training supplemented by at-home practice. The training session was shown in Figure 2. The time taken for at-home practice was monitored through participants’ practice diaries. The CG received the same melodica training after the cognitive function tests post a 10-week waiting period for ethical and motivational reasons.

The instructor group for the melodica training included one main instructor (lecturer) and seven assistants. The lecturer was a professional music instructor with expertise in piano and chorus and experienced in teaching melodica to older adults. She delivered lectures on basic music theory and guided melodic playing. The assistant instructors observed the performance of the participants and provided individual help when they could not catch up with the lecture or asked questions in person.

The program began with instructions on how to produce sound in the melodica by pressing the key while blowing the mouthpiece; over the 10 weeks, the participants practiced melodies with increasing complexity. Each session started with a finger warm-up by playing a series of tunes in a prescribed order according to the lecturer’s instructions. Participants practiced the melody they learned the week before in the first half of the training session. There was a half-time break for about 5 min, where some relaxing hand and finger exercises were introduced. After the intermission, the participants first learned new melodies by singing with the lyrics, and then with the names of the notes, while clapping their hands to the rhythm. After reviewing the melody, the participants played the melody using the melodica. Group practice was followed by the individuals practicing independently in the same facility and one-on-ones with the instructors. Over the course of the 10 intervention sessions, participants practiced a total of 16 songs and mastered a minimum of eight.

### 2.4. Cognitive function tests and questionnaires

#### 2.4.1. Daily condition questionnaire

The daily condition questionnaire was administered to participants prior to the baseline screening. It included inquiries about their history of head injury, use of psychotropic medication, and past surgeries. The questionnaire also assessed participants’ musical experience, including the type of music training, duration of formal training, and current practice frequency. In addition, participants’ engagement in social activities was assessed using a cumulative score based on three items: (a) participation in a paid job, (b) involvement in volunteer activity, and (c) not frequently being alone during the daytime. Each item was assigned a value of 1 for “yes” and 0 for “no.” The questionnaire also collected information on participants’ current monthly frequency of engagement in cognitive activities such as handcrafting, handwriting, and chorus.

#### 2.4.2. Neuropsychological assessments

To verify the intervention effects reported in previous studies, we conducted the same neuropsychological tests as in our previous melodica intervention: MMSE for screening, LMT to assess verbal memory, and DSCT, TMT, and VFTs to assess executive functions. Because these tests’ procedures were described elsewhere (Guo et al., 2021), we focus here on tests newly adopted.

Different from Guo’s study, participants were instructed to name as many vegetable (not animal) words as they could in 60 s for semantic VFT (no modification for phonological VFT, with “ka” as word-initial syllable). In addition to LMT, we administered Rey Auditory Verbal Learning Test (RAVLT) (Rey, 1964; Schmidt, 1996; Lezak et al., 2004) to determine if this study could yield similar enhancement of verbal memory as the previous 16-week melodica intervention. To minimize the practice effects (McCaffrey et al., 2001), we developed two alternative Japanese word lists for the assessment in this study (Lezak et al., 2004). We made a Japanese translation of Rey’s original word list and another equivalent English word list (Rey, 1964; Geffen et al., 1994). Some words from the English list were frequently used in English, but not in Japanese, owing to cultural and linguistic differences. We replaced these words with highly familiar semantically or phonetically similar words. Familiarity information was referenced in the Nippon Telegraph and Telephone Corporation (NTT) psycho-linguistic database “Lexical Properties of Japanese” (Amano et al., 2008). Word length and auditory familiarity were matched in the two lists (Amano et al., 2006). Combined with these 15 words in the learning list, 15 words semantically or phonetically like them were included in the 30-word list for recognition task in 2 alternative lists.

The study included five learning trials, one delayed recall trial, and one delayed recognition task. In learning trials, 15 words were given at 1 s interval. Participants were instructed to remember as many of the words as possible and recall them in any order within 1 min immediately after learning and after a 20-min interval without forewarning (delayed recall). The number of correctly recalled words was counted as the recall trial score, with a maximum possible score of 15. The total score of all five learning trials was calculated as the learning-capacity measurement. Subsequently, a recognition task was performed. Thirty words were presented verbally in random order. Participants were instructed to indicate whether the word was in the learning list by pressing the “Y” for “yes” and “N” for “no” (not from the learning list). The number of words correctly recognized was the recognition task score.

#### 2.4.3. Experimental tasks

We prepared new tests with higher sensitivity—RT tasks (Rose and Ebmeier, 2006; Wilhelm and Oberauer, 2006)—to detect subtle improvements potentially caused by the shorter training period. Compared to paper-pencil-based neuropsychological tests, RT was considered more sensitive in detecting subtle changes due to wider variation in participants and less ceiling effect.

##### 2.4.3.1. Sternberg task

**FIGURE 3:**
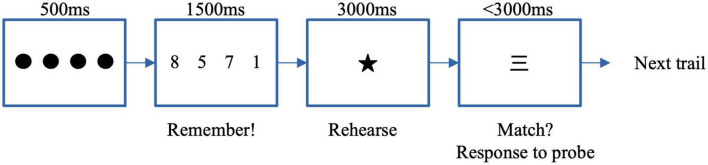
Procedures of Sternberg task in one trial.

To evaluate working memory, we administered a modified Sternberg task (Sternberg, 1966; Desmond et al., 2003). Figure 3 shows the procedure of one trial in the Sternberg task. All stimuli were presented on a 13-inch digital screen. One trial started from four fixation points (dots) presented for 500 ms. Next, in each of the four points, one Arabic numeral was presented for 1,500 ms. A single star mark was presented at the center for 3,000 ms followingly. Next, a probe (a Japanese kanji numeral) replaced the star mark. Participants were to indicate whether the probe number presented by Japanese kanji had been included in the set of four numbers presented as Arabic numerals (see Figure 3). They were instructed to indicate their answer by pressing the left key for “yes” (positive) or the right key for “no” (negative) as accurately and quickly as possible. A new trial began once the participant responded after 1,000 ms interval (pause). Participants completed 64 trials (two blocks of 32 trials each). Accuracy (% correct) and RT (ms) for every single trial was recorded.

##### 2.4.3.2. Timing task

We administered this rhythm entrainment task to measure the accuracy of participants’ timing processing and auditory-motor transformation (Wing and Kristofferson, 1973; Bernard et al., 2013). Participants were instructed to listen to tones and tap in sync with the tones. A beat of 12 tones (2,000 Hz, 20 ms) was presented with 980 ms intervals in each trial. In the first practice trial, participants were instructed to press the reaction key with their dominant hand in sync with periodic auditory tones. In the second practice trials, participants were instructed to keep tapping as consistently as possible after the auditory beat ceased, based on their memory of the beat, until they had tapped 30 times after the accompanying auditory beat had stopped. The accuracy of their “tapping based on beat memory” was measured twice; the procedure used was that of the second practice trial.

The coefficient of variance (CV) was used to quantify the performance of the rhythm entrainment task. The CV was calculated by dividing the standard deviation of the inter-tap intervals by the mean duration of the inter-tap intervals. The analyses focused on the CV for the paced continuation phase (30 taps without auditory beats).

#### 2.4.4. Additional tests

We also conducted the Grooved Pegboard Test to measure effects on complex motor coordination (Kløve, 1963),^1^ Geriatric depression scale 15 (Matsubayashi and Osawa, 1994), and a 10-item scale to investigate subjective memory (Osada et al., 1997; Kawagoe et al., 2019). These measures were mainly intended as screening or filler items before delayed recall tests and therefore, were not included in analysis. The focus of this study was on cognitive measures.

### 2.5. Data analysis

Pre-processing was conducted on the RT data in the Sternberg task. For each participant, we excluded RTs of incorrect responses and those that were longer or shorter than the individual’s mean by standard deviations (SD) of at least two. Data from 4.42% of the trials were excluded at baseline, and from 4.67% of trials were excluded in the post-intervention test. One participant was excluded from the analysis of Sternberg task performance for the low correct rate (50%), suggesting a failure in understanding the instruction.

We compared the IG and CG on demographic characteristics, cognitive functions, involvement in social and cognitive activities at baseline using two-sample *t*-test and chi-squared test to evaluate whether the pseudorandom grouping resulted in homogeneous groups using baseline data. To examine an intervention effect, we conducted a two-way mixed ANOVA with group (IG and CG) as a between-subject factor and time (pre- and post-intervention) as a within-subject factor. This ANOVA was conducted only for cognitive measures in which the IG significantly improved after training (by paired *t*-test), and an intervention effect was defined by a significant “group × time” interaction. For multiple comparison correction, we applied the Benjamini and Hochberg (1995) procedure for controlling the false discovery rate (FDR). For measures which showed intervention effects, a Pearson’s correlation analysis was performed to investigate the relation between cognitive changes and at-home melodica practice time in the IG. Furthermore, to assess the practice effect, the pre-post improvement within the CG was analyzed by paired *t*-test.

Data were analyzed using SPSS Windows version 26.0.0 (IBM Cort, 2019). Statistical significance was set at *p* < 0.05. Missing values were not filled in the analyses, but participants with missing data were discarded from the analysis of that measure (e.g., Timing task, RAVLT recognition).

## 3. Results

### 3.1. Characteristics of participants

Demographic data for the IG and CG are shown in Table 1. The data of the 30 participants (female/male = 26/4) in both the IG and CG (female/male = 24/6) were analyzed. Two-sample *t*-tests detected no significant difference between the two groups in age [IG: 73.17 (SD = 6.10); CG: 73.40 (SD = 6.05)], years of education [IG: 11.97 (SD = 2.46); CG: 12.53 (SD = 2.11)], MMSE score [IG: 28.07 (SD = 1.62); CG: 28.17 (SD = 1.91)] (see Table 1). Additionally, the two groups showed no significant differences in outcome behavioral measures, indicating that the groups were equivalent at baseline. Furthermore, no significant difference was detected between the two groups in the engagement of social or cognitive activities.

**TABLE 1:** Demographic data and engagement in social and cognitive activities for intervention and control groups at baseline.

	Intervention group	Control group	*p*-Value
Number of females/males	26/4	24/6	0.488
Age (years)	73.17 (6.10)	73.40 (6.05)	0.882
Education (years)	11.97 (2.46)	12.53 (2.11)	0.342
MMSE	28.07 (1.62)	28.17 (1.91)	0.828
Social engagement (points)	1.03 (0.93)	1.27 (0.64)	0.398
CA (monthly frequency)	4.57 (8.01)	6.65 (11.73)	0.337

Values are mean (SD, standard deviation), unless otherwise indicated. p-Values are the results of Chi-square test for the number of females/males and two sample t-test for other measures. For social participation, points represent sum of three items (yes = 1, no = 0): (1) participation in a paid job, (2) participation in volunteer activity, and (3) not frequently being alone during the daytime. MMSE, Mini-Mental Examination; CA, cognitive activities.

### 3.2. Effects of intervention and repetition

**FIGURE 4:**
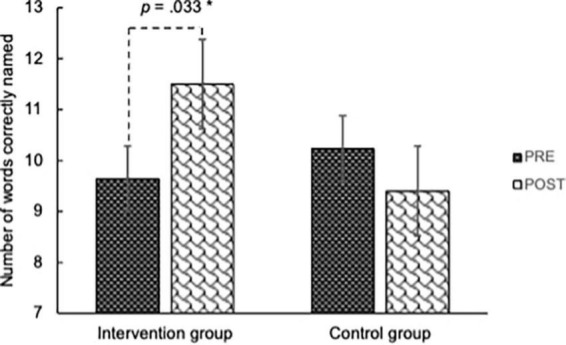
Number of words correctly named in phonological verbal fluency task. A two-way ANOVA found a significant group × time interaction: [*F*(1,58) = 6.942, *p* = 0.011, partial η^2^ = 0.107]. The main effect of time was significant only in the intervention group [*F*(1,29) = 5.023, *p* = 0.033, partial η^2^ = 0.148]. *A significant simple main effect of time in the IG (*p* < 0.05).

**FIGURE 5:**
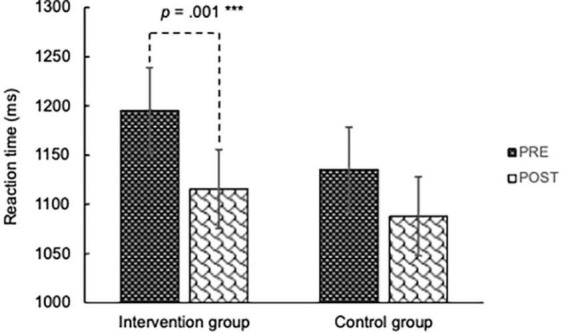
Reaction time (ms) for the Sternberg task in intervention group and control group. A two-way ANOVA found a significant group × time interaction: [*F*(1,51) = 5.292, *p* = 0.026, partial η^2^ = 0.094]. The main effect of time was only significant in the intervention group [*F*(1,25) = 15.936, *p* = 0.001, partial η^2^ = 0.389]. ***A significant simple main effect of time in the IG (*p* < 0.001).

The paired *t*-test detected significant improvement in the IG on LMT delayed recall [*t*(29) = 4.930, *p* < 0.001], DSCT [*t*(29) = 3.291, *p* = 0.003], phonological VFT [*t*(29) = 2.241, *p* = 0.033], and RT in the Sternberg task [*t*(25) = −3.992, *p* < 0.001]. Other cognitive measures showed no significant improvement in the IG. Among these four measures, the “group × time” ANOVA revealed a significant interaction on the phonological VFT [*F*(1,58) = 6.942, *p* = 0.011, partial η^2^ = 0.107] (see Figure 4). It remained significant after FDR correction (corrected *p* = 0.044). The “group × time” interaction was also significant on the RT in the Sternberg task [*F*(1,51) = 5.292, *p* = 0.026, partial η^2^ = 0.094] (see Figure 5). It was marginally significant after FDR correction (corrected *p* = 0.052). A significant “group × time” interaction was not detected in the LMT delayed recall or the DSCT.

As Table 2 shows, the IG improved 1.87 words in the phonological VFT and shortened their RT in the Sternberg task for 97.87 ms after the intervention. The IG practiced melodica at home for 1,692.86 min on average (SD = 1,312.79). Correlation between the at-home practice time and cognition improvement was not significant with either the phonological VFT (*r* = 0.209, *p* = 0.286) or the RT in the Sternberg task (*r* = −0.014, *p* = 0.949).

**TABLE 2:** Cognitive outcomes of intervention and control group.

	Intervention group (*n* = 30)		Control group (*n* = 30)		Group × time interaction		
Measures	PRE	POST	PRE	POST	*F*	*p*-Value	gCorrected *p*-value
LMT delayed recall	13.56 (6.89)	18.40 (7.62)	14.33 (8.44)	18.55 (7.84)	0.491	0.486	0.486
DSCT	58.16 (18.33)	66.47 (17.58)	62.70 (14.75)	65.57 (15.32)	3.993	0.050	0.067
TMT Part B (s)	101.70 (40.18)	93.43 (37.59)	98.33 (49.63)	86.57 (38.31)			
Semantic VFT	16.33 (4.87)	17.10 (4.66)	17.03 (4.48)	17.50 (4.45)			
Phonological VFT	9.63 (3.57)	11.50 (5.49)	10.23 (4.82)	9.40 (3.42)	6.942	0.011*	0.044*
RAVLT total learning	43.93 (10.69)	45.07 (9.56)	44.90 (9.32)	45.27 (8.57)			
RAVLT delayed recall	9.43 (3.52)	10.07 (2.88)	9.20 (3.06)	9.50 (3.21)			
RAVLT recognition	28.00 (3.17)	28.24 (1.77)	28.47 (1.36)	29.03 (3.27)			
Reaction time (ms)	1,195.33 (237.36)	1,115.46 (216.11)	1,135.33 (219.13)	1,087.87 (209.25)	5.292	0.026 *	0.052
Timing task (CV in ms)	0.19 (0.01)	0.19 (0.01)	0.19 (0.02)	0.19 (0.01)			

Values are mean (SD). The unspecified unit is a score. LMT, Logical Memory Test; DSCT, Digit Symbol Coding Test; TMT, Trail Making Test; VFT, Verbal Fluency Test; RAVLT, Rey Auditory Verbal Learning Test; Reaction time (ms), reaction time in Sternberg task; CV in ms, coefficient of variation in ms. *p < 0.05, statistically significant.

The improvement in the CG, indicative of the test-retest practice effect, was significant in several tests, including the LMT delayed recall [*t*(29) = 3.942, *p* < 0.001], DSCT [*t*(29) = 2.819, *p* = 0.009], TMT Part B [*t*(29) = −3.118, *p* = 0.004]. The practice effect was not significant on other measures.

## 4. Discussion

This study was designed to examine the effects of 10-week melodica training on cognitive function in healthy older adults. The IG and CG were matched on demographic characteristics, cognitive functions, and engagement in social and cognitive activities at baseline. A comparison was made between these two homogenous groups, with one receiving the intervention and the other serving as the control. On the one hand, it did not replicate intervention effects on the LMT delayed recall, DSCT, or TMT Part B reported in previous longer-term musical instrument RCTs. On the other hand, improvement was significant in the phonological VFT and marginally significant in the Sternberg task.

### 4.1. Improved verbal working memory

In this study, the intervention effect was significant on VFT in the phonological fluency condition and marginally significant on RT in the working memory (Sternberg) task, suggesting that verbal working memory was improved through the 10-weekly session musical instrument training intervention.

The VFT detected intervention effects with no significant practice effect, suggesting its usefulness in detecting intervention effects. To date, no music intervention studies have shown a clear improvement in the VFT. A 3-h concentrated group piano practice in older adults was reported to enhance both phonological and semantic VFT in a pilot study (Bugos and Kochar, 2017). However, the subsequent 16-week piano training intervention program did not improve the participants’ performance on the VFT compared with participants who received percussion instruction or music listening instructions (Bugos, 2019). Additionally, a 16-week melodica training did not improve participants’ performance on VFT in a previous study by our research team (Guo et al., 2021).

In children and adolescents, previous longitudinal studies have provided evidence showing the positive effects of musical training on working memory (Bergman Nutley et al., 2014; Roden et al., 2014a; Guo et al., 2018). However, non-significant improvements on working memory were also reported in music training interventions in children (Barbaroux et al., 2019). Thus, short-term musical instrument training may modestly improve working memory, if any. Consistently, our study detected working memory-related improvement, with the significant intervention effect on the phonological VFT and marginally significant intervention effect on RT in the Sternberg task. Our results suggest that healthy older adults maintain the ability to improve working memory through musical instrument training.

How can musical instrument training improve verbal working memory? Association between musical training experience and language processing has been interpreted as common acoustic processing (Besson et al., 2011, 2017). A recent study reported that music and language rely on similar working memory resources: musical stimuli produce similar working memory interference as linguistic stimuli (Fennell et al., 2021). It is also reported that both music and language processing elicited similar variations in brain electrical potentials with overall shorter onset latency for musicians than for non-musicians (Schön et al., 2004). Enhanced language processing ability was detected in dyslexic children following 18 h of musical training (Habib et al., 2016). In this study, the melodica practice improved older adults’ working memory which was presumably used in common for music processing and verbal processing. The improvement of verbal processing ability is important because it could mediate the improvement in verbal memory and executive function reported in previous studies.

### 4.2. Comparison with our Kyoto study: VFT and verbal memory measures

In our previous melodica intervention with a longer duration (16 weeks), the intervention effects were significant on verbal memory (LMT delayed recall) but not on verbal fluency (Guo et al., 2021).

For the inconsistency in VFT results, we examined differences in participants. While the mean age of participants was virtually identical in these two studies, one possible factor emerged: the participants had approximately one less year of education, based on the mean, in this study than those in our prior study. Considering that education affects phonological VF performance (Moraes et al., 2013), the 1-year difference in education may be crucial. In this study, the IG improved 2.87 words from 9.63 at baseline and the CG improved – 0.83 words from 10.23. In Guo’s study, the number correctly named changed less than one word in two groups from 12.30 (IG) and 11.63 (CG), respectively. Thus, we suggest that there may be a limit of 12 words for phonological VFT (for “ka” in Japanese), on average, for healthy older adults of approximately 73 years of age; the baseline score of participants in Guo et al. (2021) was almost the limit. Presumably, owing to a ceiling effect, the group-by-time interaction was not detected in Guo’s study. In this study, because fewer words were named at baseline (perhaps owing to the lower level of education), participants had more room for improvement. Hence, we suggest that the effect of musical instrument training on phonological VFT is more detectable for a sample with fewer years of education, although this supposition requires further investigation.

This inference is partly supported by the correlation between education and phonological VFT in this study. It is widely recognized that phonological VFT is more strongly correlated with education than age, whereas semantic VFT is more strongly correlated with age (Abe et al., 2004; Mitrushina et al., 2005). Consistent with these reports, the present results indicate that our participants with more education performed better in the phonological VFT at baseline (*r* = 0.487, *p* < 0.001). Note that the “group × time” interaction stayed significant in an ANCOVA with the years of education as a covariate [*F*(1, 57) = 5.949, *p* = 0.018, partial η2 = 0.095] in this study.

For the LMT delayed recall, both groups improved significantly after the training period, which might be due to repeating the test. These results were different from the previous study showing a significant intervention effect with a significant main effect of time only in the IG (Guo et al., 2021). One possible explanation for the inconsistency is that the interval between the pre-and post-cognitive function test was much shorter in this study than in the previous study—12 weeks on average, at least 8 weeks shorter than the previous study. As a typical memory assessment, the observed practice effects were expected in the LMT (Theisen et al., 1998; Lo et al., 2012), especially for short test-retest intervals. Practice effect was significant on story recall tasks in healthy older adults (mean age = 71.67 years old) even after a 5-year interval (Gavett et al., 2016). Hence, we recommend using alternative stories or test intervals in longitudinal studies.

The RAVLT also did not show any significant intervention effects. Unlike in the LMT, neither group improved their RAVLT performance in the post-intervention test. In previous studies, the effects of a theater art intervention for less than 10 weeks were detected on word list recall (and VFT) (Noice et al., 2004; Noice and Noice, 2008). Healthy older adult participants were trained to recall their short lines during the intervention for verbal memory training. Therefore, the word list recall (and VFT) measured a near transfer of the training (i.e., an improvement in tests, which is similar to training). In our study, verbal ability was not directly trained, and RAVLT (word list recall, i.e., context-free verbal memory) was perhaps too far from musical instrument training. Combined with the LMT results, if the melodica training intervention improves verbal memory in a longer intervention period, the improvements would be only for context-based memory (story recall), not word list recall or rote memorization.

### 4.3. Comparison with previous piano training interventions

Six months of individual piano practice improved older adults’ performance on the DSCT and TMT Part B (Bugos et al., 2007). However, the reported intervention effects were not replicated in this study. Our previous melodica intervention study with participants of similar demographic characteristics (means: 73 years of age, 13.0 years of education) did not improve their performance in TMT Part B either (Guo et al., 2021, DSCT not conducted). Therefore, one possible cause of this inconsistency is the kind of musical instrument that was used. Previous piano interventions require bimanual coordination (Bugos et al., 2007), whereas our melodica intervention only requires using the right hand. Additionally, participants’ demographic characteristics also may have affected the outcomes. The participants in the piano training intervention were approximately 3 years younger in age and had, on average, higher levels of education compared to those in this study [Bugos et al. (2007): 69.6 years old, 16.5 years of education; this study: 73.3 years old, 12.3 years of education]. It remained to be investigated which factor (education, age, or the kind of musical instrument) played the main role in the variances of intervention effects.

### 4.4. Limitation in study design: lack of active control

During the intervention period in this study, the IG gathered to undergo the musical instrument training together once per week, while the passive CG maintained their usual habits and routines. Although the two groups did not show significant differences in their engagement in social, intellectual and cognitive activities at baseline, it remained challenging to attribute the improvements solely to musical training rather than a general novice stimulation. Therefore, it is yet to be confirmed whether we can replicate the improvement in verbal working memory in the musical instrument training group compared with the active CG.

In previous music intervention studies with active controls, participants assigned to the CG listened to music during the intervention period, and no significant group-by-time interaction was detected on VFT, TMT Part A and Part B, or Stroop tasks (Bugos, 2010, 2019). Compared to musical interventions, a larger number of studies have investigated the effects of physical exercise in older adults. For the aerobic exercise IG, non-endurance programs such as light stretching have been considered as a control condition (Kramer et al., 1999; Voss et al., 2013; Tarumi et al., 2022). However, the effect of stretching was later reported on memory functions (Ruscheweyh et al., 2011; Hötting et al., 2012). Thus, physical exercise related effects might have been underestimated when compared to an active CG (for a review, see Hötting and Röder, 2013). Therefore, when implementing an active control, a three-group design may be desirable to compare the change in cognition between the IG and two CGs (active CG and passive CG).

### 4.5. Other limitations and future work

First, the intervention effects detected in this study might have been affected by the participants’ characteristics. Participants improved their phonological VFT performance, but the same-age and longer education sample did not in our previous melodica intervention (Guo et al., 2021). Although this inconsistency suggests that the intervention on the phonological VFT depends on education, in our more recent longitudinal study of over 4 years, participants returned from our Kyoto Study (more highly educated than they did in the current study). In these returned participants, those who had continued melodica practice for more than 3 years better maintained their phonological verbal fluency compared to those who had quit practice (Wang et al., in preparation)^2^. Therefore, the phonological VFT may be promising to detect effects of musical instrument practice on cognitive function in advanced aging. Another participant-related limitation is that our participants were mostly women (50 women and 10 men). Therefore, future studies should clarify whether improved verbal working memory can be generalized to men.

Second, the high correct-response rate in the Sternberg task indicated a ceiling effect, which suggests that the task was too easy for healthy older adults. We used memory sequences of only four digits (Arabic numerals); therefore, increasing the difficulty of the task by increasing the number of digits may enable a more precise assessment of the improvement of working memory.

Third, the intervention period seemed too short, which was suggested by two aspects: (1) the test-retest practice effect was significant in several measures. (2) The IG showed no significant improvement on the timing task. The latter suggests that the 10-week melodical training was insufficient for improving auditory-motor transformation in musically untrained older adults. We conducted this study in collaboration with the Osaka local government. We set an intervention period for 10 weeks to finish all procedures of this project (participant recruitment, pre-and post-intervention/waiting test, intervention, and providing the same melodica lessons to the waiting group after the post-test) within one financial year. However, the results suggested the need for an extended interval between screenings to capture more rigorous intervention effects, which could be justified by implementing a longer training period in the same study. A longer training period might also be beneficial for participants to enhance their music performance skills and improve their related cognitive functions, especially for those who had difficulty keeping up with group lessons (see Kakihara et al., in revision)^3^. Fortunately, our informal assessment found the participants’ high motivation for continuing making music after the research program.

## 5. Conclusion

This study did not find a clear-cut intervention effect on LMT delayed recall, DSCT, and TMT Part B which were found in previous studies. The shorter intervention period may yield a (1) subtler intervention effect and (2) stronger test-retest effect which may disrupt detecting intervention specific-effects. A longer training period may be less subject to the test-retest practice effect and likely show a more clear-cut intervention effect, although it will be warranted by a direct manipulation of the period of training.

Nevertheless, the intervention effects were detected significant on the phonological VFT and marginally significant on RT in the Sternberg task, indicating the improvement in verbal working memory after this 10-week musical instrument training program. To the best of our knowledge, this study is the first to demonstrate the improved phonological verbal fluency and the shortened RTs for the Sternberg task following musical intervention. The improvement of verbal working memory may be an early and essential cognitive improvement induced by participation in the instrumental training program because it has the potential to mediate further improvement of verbal memory, processing speed, and executive function.

## Data availability statement

The data that support the findings of this study are available from the corresponding author upon reasonable request.

## Ethics statement

The studies involving human participants were reviewed and approved by the Ethics Committee of Kyoto University. The patients/participants provided their written informed consent to participate in this study.

## Author contributions

XW performed the research, analyzed the data, writing—original draft, and writing—review, and editing. TS, MY, and MK performed the research, review, and editing. TT programmed the cognitive function tests. SI performed the research. KS designed the research and supervision, recruited participants, and wrote, reviewed, and edited the manuscript. KS agreed to be accountable for all aspects of the work, ensuring that questions related to the accuracy or integrity of any part of the work were appropriately investigated and resolved. All authors contributed to the article and approved the submitted version.

## Footnotes

1
https://lafayetteevaluation.com/products/grooved-pegboard

2 Wang, X., Yamashita, M., Guo, X., Osawa, C., and Sekiyama, K. (in preparation). Effects of musical instrument practice on cognitive reserve in older adults: A three-year follow-up study.

3 Kakihara, M., Wang, X., Iwasaki, S., Soshi, T., Yamashita, M., and Sekiyama, K. (in revision). The association between performance skills and cognitive improvement in a 10-week musical instrument training for older adults. *Psychol. Music. Rev*.
